# Differential Dynamical Pattern of Regional Homogeneity in Bipolar and Unipolar Depression: A Preliminary Resting-State fMRI Study

**DOI:** 10.3389/fpsyt.2021.764932

**Published:** 2021-12-13

**Authors:** Fuping Sun, Zhening Liu, Jun Yang, Zebin Fan, Jie Yang

**Affiliations:** National Clinical Research Center for Mental Disorders, Department of Psychiatry, The Second Xiangya Hospital of Central South University, Changsha, China

**Keywords:** bipolar depression, unipolar depression, dynamics, regional homogeneity, machine learning

## Abstract

**Background:** Bipolar depression (BD) and unipolar depression (UD) are both characterized by depressive moods, which are difficult to distinguish in clinical practice. Human brain activity is time-varying and dynamic. Investigating dynamical pattern alterations of depressed brains can provide deep insights into the pathophysiological features of depression. This study aimed to explore similar and different abnormal dynamic patterns between BD and UD.

**Methods:** Brain resting-state functional magnetic resonance imaging data were acquired from 36 patients with BD type I (BD-I), 38 patients with UD, and 42 healthy controls (HCs). Analysis of covariance was adopted to examine the differential pattern of the dynamical regional homogeneity (dReHo) temporal variability across 3 groups, with gender, age, and education level as covariates. *Post-hoc* analyses were employed to obtain the different dynamic characteristics between any 2 groups. We further applied the machine-learning methods to classify BD-I from UD by using the detected distinct dReHo pattern.

**Results:** Compared with patients with UD, patients with BD-I demonstrated decreased dReHo variability in the right postcentral gyrus and right parahippocampal gyrus. By using the dReHo variability pattern of these two regions as features, we achieved the 91.89% accuracy and 0.92 area under curve in classifying BD-I from UD. Relative to HCs, patients with UD showed increased dReHo variability in the right postcentral gyrus, while there were no dReHo variability differences in patients with BD-I.

**Conclusions:** The results of this study mainly report the differential dynamic pattern of the regional activity between BD-I and UD, particular in the mesolimbic system, and show its promising potential in assisting the diagnosis of these two depression groups.

## Introduction

Bipolar disorder, also known as manic depression, is characterized by the presence of recurring manic or hypomanic and depressive episodes. Bipolar depression (BD), depressive episodes of bipolar disorder, last considerably longer than manic episodes of bipolar disorder (1). The depressive episode is an overlapping characteristic between bipolar disorder and unipolar depression (UD), which often leads to misdiagnosis of BD and UD (2). Studies report that nearly 69% of patients with BD have been diagnosed as UD (3), which can easily lead to inappropriate treatment and poor prognosis (4). Thus, how to differentiate BD from UD has been a longstanding clinical challenge in psychiatry.

Although the overall clinical phenomenology and molecular mechanisms (5, 6) of BD and UD can be extremely similar, it is acknowledged that their clinical features were subtly different at more fined granularity after further investigations. Previous epidemiology studies have documented patients with BD are likely to report prior psychoactive medications, lifetime psychotic symptoms, nicotine abuse, younger age at illness onset, and first hospitalization (7), while patients with UD tend to have more pain sensitivity, somatic complaints, and insomnia. However, these clinical findings were obtained from self-report and subjective assessment. It is still difficult to benefit from them for aiding in distinguishing BD and UD more accurately and effectively (8).

Recently, accumulating neuroimaging studies have probed the neurobiological mechanisms of BD and UD (9, 10), aiming at pursuing objective and reliable biomarkers for distinguishing these 2 diseases. Among various neuroimaging analysis methods, regional homogeneity (ReHo) is one of the classic methods to measure brain regional activity under resting-state (11), which has been widely employed in studies of investigating differential and similar regional activity patterns between BD and UD. For example, Yao et al. (12) have highlighted that ReHo in the left temporal lobe showed significant differences between BD and UD. Liang et al. (13) have manifested a marked ReHo difference in the thalamus between BD and UD. Liu et al. (14) have documented that the ReHo differences between BD and UD extend to widespread brain areas. It was reported that, compared to patients with UD, patients with BD exhibited increased ReHo in the right dorsal anterior insular, right middle frontal gyrus and cerebellum lobe, as well as decreased ReHo in the right parahippocampal gyrus and right anterior insular. With a growing number of relevant research have being conducted, a critical problem also began to emerge. That is although substantial studies have investigated the neurobiological mechanism of these 2 diseases, even using the same index (e.g., ReHo), their findings were varied. One of the potential reasons for this inconsistency may be that those studies recruited different subtypes of patients with BD, and the heterogeneity of mixed subtypes of patients with BD may hinder the progress of detecting reliable distinctive biomarkers.

Given that human brain activity is time-varying and dynamic (15), neuroimaging researchers have proposed analysis methods of the brain dynamic pattern that related to some classic static indices, such as dynamic ReHo (dReHo) (16), dynamic functional connectivity (17), and dynamic low-frequency fluctuation fractional amplitude (18). These advanced computation analysis methods have gradually been applied in the neuroimaging study of mental illness (19–21), showing their potential advantages in assisting diagnosis. Previous studies have employed the detected dynamical pattern alterations as features and found that it can improve the diagnostic accuracy of major depressive disorder by about 15 percentage points than the static pattern (22). Among various dynamical indices, dReHo temporal variability indicts the dynamical regional activity pattern (23), which aims to calculate the temporal variability of the ReHo index on a certain timeseries after parcellated the whole timeseries into a series of windows. However, there are limited studies that have employed the dynamic analysis method to investigate the brain dynamics of regional activity between BD and UD, especially the study of recruiting only one BD subtype.

Based on the limitations of the prior studies and considering there were very few studies that have investigated the dynamic pattern of regional neural activity in patients with BD and UD, especially in a pure dataset that all patients with BD belong to the same subtype of bipolar disorder. We measured the whole-brain dReHo at voxel-wise to investigate the abnormal pattern of dynamic regional activity in BD type I (BD-I) and UD. We hypothesized that BD-I and UD shared some dReho variability patterns, but also have their specific abnormal dReho variability profiles, which may help to distinguish BD-I from UD. We also hypothesized that in these 2 diseases, the detected brain regions with abnormal dynamic patterns of regional activity may partially be consistent with previous studies using the static ReHo, considering the homogeneity of these 2 indices.

## Methods

### Ethic Statement

Permission to undertake this study was granted by the Ethics Committee of the Second Xiangya Hospital, Central South University, Changsha, China. Prior to the examination, all participants were right-handed native Chinese speakers, and they were provided a written informed consent form after a full-written and verbal explanation of the study by 2 licensed psychiatrists. All study procedures were conducted in strict accordance with the Declaration of Helsinki.

### Participants

The study recruited 99 patients, including 51 patients with BD-I, 48 patients with UD from the psychiatry department of Second Xiangya Hospital of Central South University. All patients meet the Diagnostic and Statistical Manual of Mental Disorders, Fourth Edition (known as DSM-IV) criteria for BD-I and UD according to the diagnostic assessment using the Structured Clinical Interview for DSM-IV Patient Edition (SCID-P) were interviewed by 2 experienced psychiatrists. The Exclusion criteria for the patients were as follows: (1) <18 years old or >48 years old; (2) previous electroconvulsive therapy and any other contraindications to MRI; (3) history of alcohol or substance abuse except nicotine; (4) chronic neurological disorders or debilitating physical illness; (5) benzodiazepine treatment, if any, stopped <24 h before scanning.

A total of 46 healthy controls (HCs) were recruited from local communities and schools, and they were assessed using the Structured Clinical Interview for DSM-IV Axis I Disorders, Research Version, Non-patient Edition (SCIDI/NP). The inclusion and exclusion criteria for HCs were the same as those for patients except that they did not meet the DSM-IV criteria for any mental disorders.

### Assessment of Clinical Symptoms

Before undergoing Magnetic Resonance Imaging (MRI), all patients were screened with the 17-item Hamilton Depression Rating Scale (HAMD) (24), Hamilton Anxiety Rating Scale (HAMA) (25), Young Mania Rating Scale (YMRS) (26) and Brief Psychiatric Rating Scale (BRPS) (27) during the 3 days before the imaging session in order to estimate the clinical symptoms. The inclusion criterion was a total HAMD score of ≥17 and a total YMRS score of <6 for the patients with BD-I, and a total HAMD score of ≥17 for the patients with UD.

### Data Acquisition and Pre-processing

In this study, all participants were scanned on a Philips Gyroscan Achieva 3.0 T using a gradient-recalled echo-planar imaging pulse sequence. The specific scanning sequence with the following parameters: 36 slice, matrix = 64 ×64, Repetition Time (RT) = 2,000 ms, flip angle (FA) = 900, echo time (TE) = 30 ms, gap = 0 mm, slice thickness = 4 mm, 250 total volumes. During the scan, all subjects kept closed their eyes and did not fall asleep.

Data preprocessing was performed using the Data Processing and Analysis for (Resting-State) Brain Imaging (DPABI) toolbox (28). The first 10 volumes of functional time points allowed the participants to adjust for magnetic saturation delay. The left 240 volumes were included in the following analyses: slice timing correction, motion realignment, spatial normalization with the brain template of Montreal Neurologic Institute (MNI), linear detrending, and band-pass filter (0.01–0.08 HZ). Nuisance covariates including 12 head motion parameters (6 head motions and their temporal first derivatives), global mean signals, white matter, and cerebrospinal fluid (CSF) signals were regressed out from the blood oxygen level-dependent (BOLD) signals. In line with our prior study, displaced volumes (framewise displacement > 0.5 mm) were interpolated by nearest-neighbor interpolation (29). The exclusion criteria for sample selection included: (1) Head motions larger than a 2.5-mm translation or 2.5° rotation in any direction; (2) More than 48 volumes were scrubbed (i.e., >20% of the acquired analysis); (3) Functional MRI (fMRI) data failed to normalize to MNI space which is visually inspected by an experienced data analyst. After quality control, 36 patients with BD-I, 38 patients with UD, and 42 HCs were satisfied with head motion. No significant difference was found in framewise displacement (total number of interpolated volumes) across all 3 groups (*F*_2,125_ = 0.960, *P* = 0.386).

### Temporal Variability of the dReho Calculation

A sliding window approach was used to calculate the dReho by using the Dynamic Brain Connectome (DynamicBC) toolbox (30). In this study, we used a window length of 50 TRs (100 s) to calculate the temporal variability of dReho, which was accorded with the recommendation of previous studies (100 s ≥ 1/0.01) (31). The time series was comprised of 240 TRs (480 s), and the window was shifted by 1 TR (2 s). The full-length time series was then divided into 191 windows for each subject. We obtained a Reho map for each sliding window. We then computed the coefficient of variation of 191 dReho maps across all sliding windows to explore the temporal variability of the 3 groups, and the dynamical ReHo temporal variability map for each subject was generated. Finally, the dReHo map was smoothed with FWHM = 6 mm.

### Statistical Analyses

We conducted analyses to test the demographic and clinical characteristics of the 3 groups by using SPSS 21.0 software. Differences in age and education years were analyzed with a one-way analysis of variance (ANOVA). Differences in the duration of illness, age of onset, the chlorpromazine (CPZ)-equivalents (32) medication dosage, and clinical scale scores, including the HAMD, HAMA, YMRS, and BRPS between 2 patient groups were compared by two-sample *t*-test. At last, we used the chi-square test to calculate differences in gender across 3 groups and differences in medication information between 2 patient groups.

The dReho variability among the 3 groups was performed using Statistic Parameter Mapping 8 software (www.fil.ion.ucl.ac.uk/spm). A one-way analysis of covariance (ANCOVA) was carried out to compare the dReho variability among the 3 groups at voxel-wise with age, gender, and education years as nuisance covariates. Then, by applying the significant voxels that survived in ANCOVA analysis as the mask, *post-hoc t*-tests were performed between any two groups. The threshold was set at voxel-level *p*_voxel_ < 0.005 and cluster size > 26 (AlphaSim corrected, *p*_cluster_ < 0.05).

### Machine Learning Analyses

The pattern recognition analysis was further adopted to test whether the detected differential dynamical pattern between BD-I and UD carries sufficient illness-specific information that can discriminate these 2 disorders. We extracted the dReho temporal variability of regions that show significant differences between BD-I and UD and conducted the pattern recognition analysis by using the support vector machine (SVM) toolkit libsvm (https://www.csie.ntu.edu.tw/~cjlin/libsvm/).). In line with our prior study (33), we set the kernel function of the SVM as the sigmoid type, cost c = 10, and other all related parameters were default set to trade-off learning and extensibility (g = 1/number of all features, and coef = 0). The Leave-One-Out-Cross Validation was employed to evaluate the general performance. The average prediction accuracy, area under curve (AUC) (34), as well as the true positive rate for BD-I and UD were calculated to evaluate the performance.

### Correlation With Symptom Scores

We conducted the correlation analysis between the dReHo variability of brain regions with significant omnibus differences with HAMD, HAMD, YMRS Scores, as well as duration of illness of BD-I and UD.

### Validation Analyses

We used one additional sliding window lengths of 80 TRs (160 s) to validate our main findings with using window lengths of 50 TRs (100 s).

## Results

### Clinical and Demographic Characteristics

There were no significant differences among the 3 groups in terms of gender (*p* = 0.742), age (*p* = 0.225), years of education (*p* = 0.093) and mean framewise displacement (FD) (*p* = 0.386). Additionally, HAMD (*p* = 0.956), HAMA (*p* = 0.928), YMRS (*p* = 0.303), and the illness duration (*p* = 0.349) also showed no differences between the patient groups. Notably, the onset age of BD-I is earlier than UD (*p* = 0.006), and total score of BPRS with BD-I is higher than UD (*p* = 0.042). All data were summarized in Table 1. However, we did not observe differences in BD-I and UD when we further classify BPRS into 5 subcategories (see Supplementary Material 1).

**Table 1:** Cohort demographics and clinical characteristics.

**Characteristics**	**BD-I (***n*** = 36)**	**UD (***n*** = 38)**	**HCs (***n*** = 42)**	***F/T*** **/χ^2^**	***P***
**Sociodemographic**					
Gender (M/F)	19/17	20/18	19/23	0.597	0.742^a^
Age (years)	27.14 ± 7.64	30.21 ± 8.39	28.69 ± 6.74	1.514	0.225^b^
Age range (years)	18–45	18–47	20–43	N/A	N/A
Education (years)	13.44 ± 3.02	12.55 ± 2.91	13.95 ± 2.67	2.427	0.093^b^
Age on onset (years)	22.50 ± 6.12	27.41 ± 8.57	N/A	2.818	**0.006** ^c^
Duration of illness (months)	56.10 ± 61.20	43.28 ± 70.78	N/A	0.827	0.411^c^
Mean FD	0.15 ± 0.07	0.15 ± 0.07	0.13 ± 0.05	0.960	0.386^b^
**Questionnaires**					
HAMD	21.56 ± 4.12	21.50 ± 4.48	N/A	0.055	0.956^c^
HAMA	16.69 ± 8.31	16.87 ± 8.79	N/A	0.091	0.928^c^
YMRS	1.77 ± 1.96	2.34 ± 2.65	N/A	1.038	0.303^c^
BPRS	33.45 ± 8.47	29.82 ± 5.74	N/A	2.086	**0.042** ^c^
**Medication**					
CPZ-equivalents (mg)	151.24 ± 190.80	31.51 ± 87.96	N/A	3.224	**0.002** ^c^

a *Chi-square test (two-tailed)*.

b *ANOVA*.

c *t-test (two-tailed)*.

*Values are presented by mean ± standard deviation. The bold values provided in the table are highlighted*.

*BD-I, bipolar type I depression; UD, unipolar depression; HCs, healthy controls; FD, framewise displacement; HAMD, Hamilton Depression Rating Scale; HAMA, Hamilton Anxiety Rating Scale; YMRS, Young Mania Rating Scale; BPRS, Brief Psychiatric Rating Scale*.

We observed differences in CPZ-equivalent between 2 patient groups, and patients with BD-I took more antipsychotics (see Table 1) and more mood stabilizer, but less antidepressants than patients with UD. The details of CPZ-equivalent calculation were documented in Supplementary Material 2, and the details of medication information were recorded in Supplementary Material 3.

### Temporal Variability of the dReho

**Figure 1:**
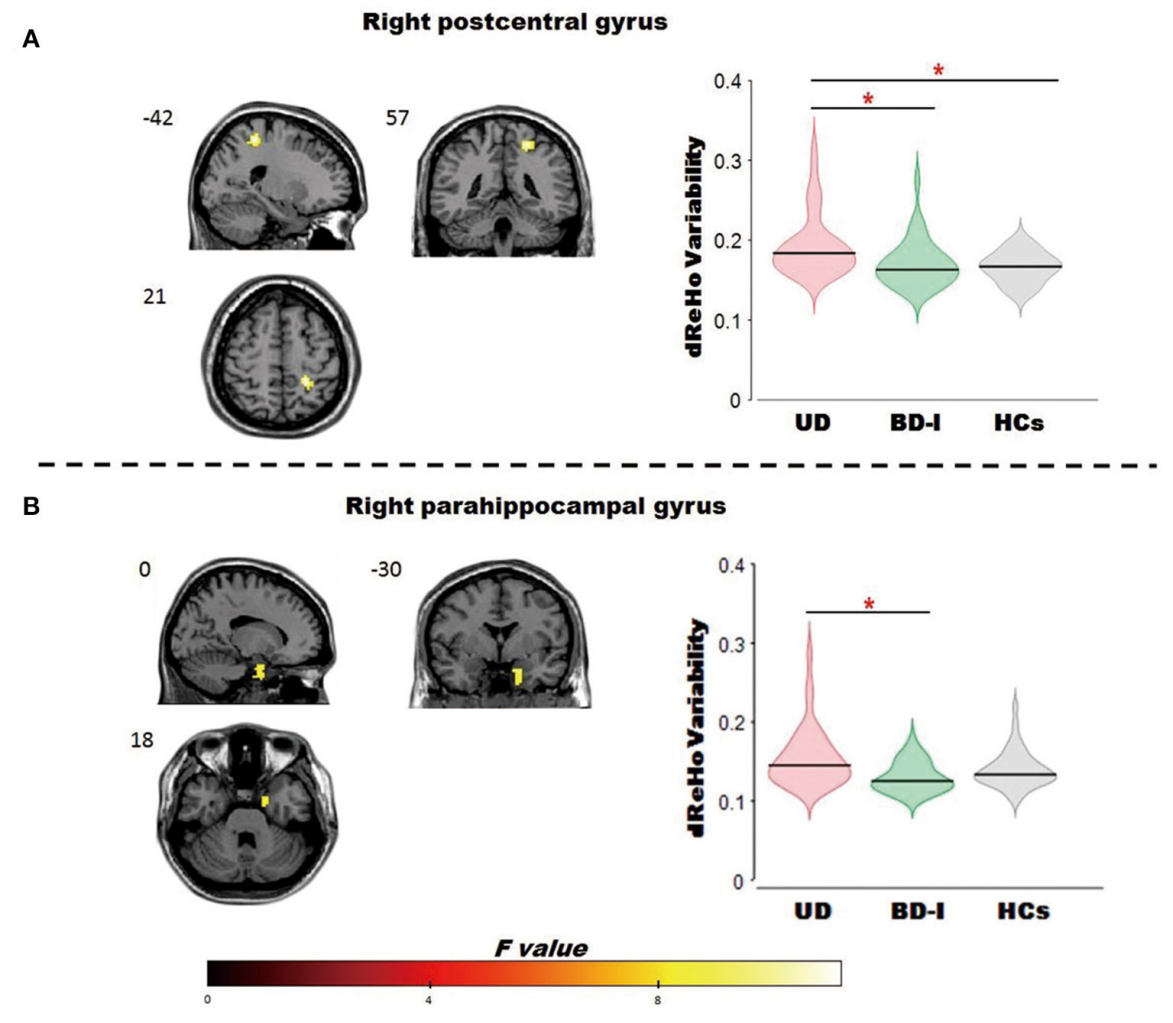
Brain regions showed significant omnibus differences of the dReHo variability among BD-I, UD and HCs groups. **(A)** Compared to patients with UD, both patients with BD-I and HCs showed a decreased dReho variability in the right postcentral gyrus; **(B)** compared to patients with UD, patients with BD-I showed a decreased dReho variability in the right parahippocampal gyrus. *represents *p* < 0.05. dReho, dynamic regional homogeneity; BD-I, bipolar type I depression; UD, unipolar depression; HCs, healthy controls.

ANCOVA revealed a significant dReHo temporal variability difference in the right postcentral gyrus [MNI (x = 21, y = −42, z = 57); *F*_2,125_ = 9.00] among the 3 groups. *Post-hoc t*-tests revealed, compared with patients with UD, both patients with BD-I (*t* = −3.46) and HCs (*t* = −4.2) showed significantly decreased dReHo variability in the right postcentral gyrus. While the difference in the right postcentral gyrus between patient with BD-I and HCs is moderate, did not survive after AlphaSim correction (all see Figure 1; Table 2).

**Table 2:** Brain regions with significant dReho difference among the three groups.

**One-way ANOVA**						***F***	***Post-hoc*** **analysis**	
**Brain region**	**MNI**			**BA**	**Voxels**		**Comparisons**	***T***
	***X***	***Y***	***Z***					
R_Postcentral gyrus	21	−42	57	40	42	9.00	BD-I < UD	−3.46
							HCs < UD	−4.20
R_Parahippocampal	18	0	−30	28	40	7.00	BD-I < UD	−3.72
gyrus								

*BD-I, bipolar type I depression; UD, unipolar depression; HCs, healthy controls; dReho, the dynamic regional homogeneity; R, right; MNI, montreal neurological institute, BA, brodmann area*.

ANCOVA revealed a significant dReHo temporal variability difference in the right parahippocampal gyrus [MNI (x = 18, y = 0, z = −30); *F*_2,125_ = 7.00] among the 3 groups. *Post-hoc t*-tests revealed, compared to patients with UD, patients with BD-I (*t* = −3.72) showed significantly decreased dReHo variability in the parahippocampal gyrus (all see Figure 1; Table 2).

### Machine Learning Analyses

**Figure 2:**
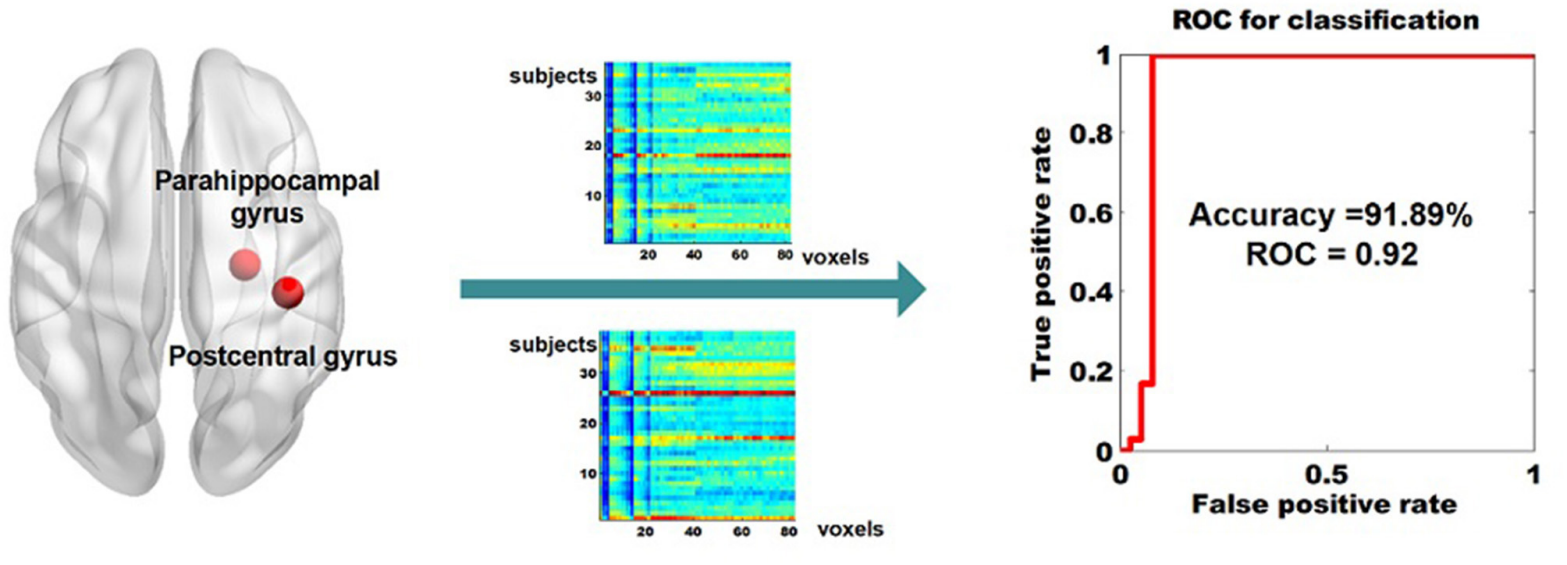
Classifying BD-I from UD by using the dReHo variability of the right parahippocampal gyrus and the right parahippocampal gyrus as features. dReho, dynamic regional homogeneity; BD-I, bipolar type I depression; UD, unipolar depression.

The dReho temporal variability in the right postcentral gyrus and in the right parahippocampal gyrus were extracted (a total of 82 voxels, i.e., 82 features) and the subsequent pattern recognition analysis was conducted. We achieved classification results with average accuracy, AUC, as well as the true positive rate for BD-I and UD, of 91.89, 0.92, 100, and 84.2%, respectively. The receiver operator characteristics of the diagnostic classification were displayed in Figure 2.

### Correlation With Symptom Scores

We did not find any significant associations between dReHo variability in the right postcentral gyrus and the right parahippocampal gyrus with HAMD, HAMA, YMRS, duration of illness. The details were presented in Supplementary Material 4.

### Validation Results

Except for the consistent right parahippocampal gyrus [MNI (x = 18, y = 3, z = −27); *F*_2,125_ = 9.62], we also observed the omnibus dReHo differences in left postcentral gyrus [MNI (x = −45, y = −27, z = 39); *F*_2,125_ = 9.31] with using 80TRs. It should be noted that the dReHo differences in the right postcentral gyrus with using 80TRs did not meet the significance threshold of AlphaSim correction, as the cluster size <26. In order to make the generated results with using 2 different window sizes more comparable, we also included the details of right postcentral gyrus in Table 3. Nevertheless, we observed the dReHo differences in left postcentral gyrus with using 80TRs. Therefore, on the whole, the results generated from 80TRs were essentially in agreement with our main findings with 50TRs.

**Table 3:** Brain regions showing significant omnibus differences in dReHo variability by using 80 TRs (160 s) as sliding window size.

**One-way ANOVA**						***F***	***Post-hoc*** **analysis**	
**Brain region**	**MNI**			**BA**	**Voxels**		**Comparisons**	***T***
	***X***	***Y***	***Z***					
L_Postcentral gyrus	−45	−27	39	40	31	9.31	BD-I < UD	−3.76
							HCs < UD	−4.51
R_Parahippocampal	18	3	−27	28	48	9.62	BD-I < UD	−4.35
gyrus								
R_Postcentral gyrus	24	−45	57	40	20	7.52	BD-I < UD	−3.47
							HCs < UD	−3.60

*BD-I, bipolar type I depression; UD, unipolar depression; HCs, healthy controls; dReho, dynamic regional homogeneity; R, right; MNI, montreal neurological institute, BA, brodmann area*.

## Discussion

We observed that patients with BD-I showed decreased dReHo variability in the right postcentral gyrus and right parahippocampal gyrus compared with patients with UD. Most notably, their dynamical differential patterns showed a promising potential in assisting diagnosis, with 91.89% accuracy and 0.92 AUC. Compared with HCs, patients with UD showed increased dReHo in the right postcentral gyrus, while no differences were observed in the comparison between patients with BD-I and HCs. To our knowledge, this is the first study to investigate the dynamical regional activity across BD-I, UD, and HCs 3 groups by using the dReHo temporal variability index, which may extend our understanding of the neurophysiology underlying BD-I and UD from the dynamical perspective.

Compared with patients with BD-I, patients with UD showed higher dReHo variability of the postcentral gyrus. The postcentral gyrus locates in the primary and secondary somatosensory cortex (33, 35), which plays a crucial role crucial role in the integration of spatial coding of somatosensory information (36), sensory discriminative dimension of pain (35), especially in the visceral and cutaneous pain (37, 38). A prior study has highlighted its activity correlated with different features of their pain behavior as well as the magnitude of the brain responses elicited by experimental tonic heat stimuli (39). Activation of the postcentral gyrus may also play an important role in facilitating the occurrence of pain in depression. It was reported (39) that the major depressive disorder patients with pain showed abnormal higher activation in the postcentral gyrus than major depressive disorder patients without pain, and there were no HAMD scores differences between these 2 patient groups. We speculated that the abnormal higher dReho variability of the postcentral gyrus in patients with UD relative to patients with BD-I may be associated with the higher pain sensitivity and somatic complaints in UD than BD, which was reported in previous epidemiology studies (40).

In addition, patients with BD-I showed decreased dReHo variability in the parahippocampal gyrus compared with patients with UD. The parahippocampal gyrus locates in the limbic system, has multiple connections with the hippocampus and amygdala, which plays an important role in emotional regulation. This finding may collaborate with our prior work and highlight there is a differential dynamic pattern in the mesolimbic system between BD-I and UD (41). Patients with BD-I tend to downregulate the sensitivity of the mesolimbic system due to having suffered more emotional states (40). Previous neuroimaging studies have reported the differential pattern of the parahippocampal gyrus between UD and BD, in terms of microstructure (42), anatomical, and activity. For example, there was a differential hippocampal synaptic pathology between bipolar disorders and UD, with the mRNAs being reduced in hippocampal CA4 and parahippocampal gyrus but no alterations in complex in mRNAs were found in UD (40). A prior study (42) has documented the decreased gray matter volume of the parahippocampal gyrus in BD compared to UD. Consistent with the current study, Liu et al. (14) have detected those patients with BD showed decreased ReHo in the parahippocampal gyrus compared with patients with UD. It collaborates with our current study and supports that there was a differential ReHo pattern of the parahippocampal gyrus between UD and BD from both static and dynamical perspectives, and future studies should shed a look into the power of the combination of these 2 indices in distinguishing UD from BD.

In the current study, the pattern recognition analysis employed the dynamical pattern in the postcentral gyrus and parahippocampal gyrus as features and achieved pretty good performance with 91.86% accuracy and 0.92 AUC. The accuracy is higher than 80%, this number is thought to be a clinical useful threshold in the consensus report of the APA work group on neuroimaging markers of psychiatric disorders (43). It may show the differential dynamic pattern of the regional activity in these two regions has promising potential in assisting the diagnosis of BD-I and UD. Our prior study only recruited the dynamical regional pattern of the putamen as features and achieved around 75% accuracy (41). Combining the machine-learning results of these two studies, we may speculate the following 2 points. First, the dynamical regional activity pattern of the mesolimbic system can be treated as a promising biomarker to classify BD-I from UD. Second, the dynamical regional activity pattern of the postcentral gyrus also carried extra useful information for aiding the diagnosis.

In the current study, no differences were detected between patients with BD-I and HCs. We should admit the current study is a very preliminary one to investigate the dynamical region activity between BD-I and UD. Studies of the dynamical regional activity in BD-I and UD are very lacking, or even none. The absence of group differences in the dReHo pattern between BD-I and UD is complex, which may be due to many factors. For example, our modest sample size cannot afford enough power to detect more results, or the dReHo variability is a reserved index and treating stable extremum numbers conservatively, but more favor ups and downs regional activity. However, the ups and downs regional activity patterns are relative rarely under resting-state without stimulations.

The present study has several limitations. First, we did not collect the relevant information about the duration of UD before the patients converted to BD diagnosis. We tracked the symptom trajectory of the patients with UD from data collection to submission of this manuscript, but did not find any cases of conversion from UD to BD-I during this time period. However, it is not possible to know if these patients will convert from UD to BD-I in the future. Second, the 2 patient groups differ in drugs, therefore, we cannot rule out the possible confounding effects of drugs on the dReHo analysis. Third, our study is preliminary, the results were only survived the relatively loose AlphaSim-correction, rather than the stricter FDR or FWE correction. Future studies of large samples and untreated patients may help to confirm the power of our results in distinguishing BD-I from UD.

## Conclusion

This study used the dReHo variability index to measure the dynamical regional activity pattern in BD-I, UD, and HCs 3 groups. We observed that patients with UD showed increased dReHo in the right postcentral gyrus and right parahippocampal gyrus compared with patients with UD, and increased dReHo in the right postcentral gyrus compared with HCs. The differential pattern of dReHo variability between BD-I and UD may relate to the differential subtle clinical symptoms between these 2 diseases. Combining the results of our prior work (41), we speculate that the differential dynamic regional activity pattern detected between BD-I and UD, especially in the mesolimbic system, may assist diagnosis and expand our understanding of the neurobiological mechanisms of these two diseases.

## Data Availability Statement

The original contributions presented in the study are included in the article/Supplementary Material, further inquiries can be directed to the corresponding author.

## Ethics Statement

The studies involving human participants were reviewed and approved by the Ethics Committee of the Second Xiangya Hospital, Central South University, Changsha, China. The patients/participants provided their written informed consent to participate in this study.

## Author Contributions

ZL and JiY designed the study. FS, ZF, and JuY acquired the data. FS and JiY analyzed the data and wrote the article. All authors contributed, approved the final manuscript, were involved in developing, editing, reviewing, and providing feedback for this manuscript and have given approval of the final version to be published.

## Funding

This work was supported by a grant from the National Natural Science Foundation of China (82071506 to ZL) and the Natural Science Foundation of Hunan Province, China (2021JJ40884 to JiY).

## Conflict of Interest

The authors declare that the research was conducted in the absence of any commercial or financial relationships that could be construed as a potential conflict of interest.

## Publisher's Note

All claims expressed in this article are solely those of the authors and do not necessarily represent those of their affiliated organizations, or those of the publisher, the editors and the reviewers. Any product that may be evaluated in this article, or claim that may be made by its manufacturer, is not guaranteed or endorsed by the publisher.
