# Supplementary material for: Differential Dynamical Pattern of Regional Homogeneity in Bipolar and Unipolar Depression: A Preliminary Resting-State fMRI Study
Source: Front Psychiatry. 2021 Dec 13;12:764932. doi: 10.3389/fpsyt.2021.764932 (PMC8710770; doi:10.3389/fpsyt.2021.764932)
Supplement: Supplementary file 1 [file Data_Sheet1.doc]

**Supplemental S1.**

**Table S1.** **Details information of the subscale scores of BPRS.**

| **Characteristics** | BD-I **(n=36)** | **UD (n =38)** | **HCs (n =42)** | ***T*** | ***P*** |
| --- | --- | --- | --- | --- | --- |
| ***BPRS*** |  |  |  |  |  |
| Hostility Scores | 3.42±1.46 | 3.74±1.27 | N/A | 0.394 | 0.317 |
| Anxiety Scores | 11.61±5.11 | 11.52±3.12 | N/A | 8.260 | 0.932 |
| Activation Scores | 3.64±1.51 | 3.82±1.06 | N/A | 0.505 | 0.615 |
| Psychosis Scores | 3.75±1.20 | 4.10±1.03 | N/A | 1.335 | 0.177 |
| Withdrawal Scores | 6.11±2.47 | 6.24±2.25 | N/A | 0.127 | 0.819 |

**Note:** two-tailed *t*-test. Values are presented by mean ± standard deviation.

**Abbreviations:** BD-I, bipolar type I depression; UD, unipolar depression; HCs, healthy controls; BPRS, Brief Psychiatric Rating Scale; Hostility Scores includes the item 10, 11, and 14; Anxiety Scores includes the item 1, 2, 5, and 9; Activation Scores includes the item 6, 8, 7, and 17; Psychosis Scores includes the item 4, 12, and 15; Withdrawal Scores includes the item 3, 13, and 16.

**Supplemental material S2. The chlorpromazine (CPZ)-equivalents details.**

The following table listed the CPZ equivalents of each antipsychotic drug taken by study participants (1).

**Table S2.** **Chlorpromazine (CPZ)-equivalents details**.

| Chlorpromazine dose | Second-generation antipsychotics | Equivalents dose |
| --- | --- | --- |
| 100 mg/d ≈ | Aripiprazole 4 (3.6) mg/d | |
|  | Clozapine 120 (107) mg/d | |
|  | Haloperidol 1.6 mg/d | |
|  | Olanzapine 3 (3.6) mg/d | |
|  | Quetiapine 60 (88.9) mg/d | |
|  | Risperidone 0.8 (1.4) mg/d | |
|  | Ziprasidone 16 (28.4) mg/d | |
|  | Rulasidone 16 (14.2) mg/d | |
|  | Sulpiride 200 mg/d | |
|  | Amisulpride 200 mg/d | |

1. S. Leucht, M. Samara, S. Heres, M. X. Patel, S. W. Woods and J. M. Davis Dose equivalents for second-generation antipsychotics: the minimum effective dose method*. Schizophr Bu*ll. (2014) 40:314-26. doi:10.1093/schbul/sbu001

**Supplemental S3.**

**Table S3.** **Details of Medication information.**

| **Characteristics** | **BD-I (n=36)** | **UD (n =38)** | **HCs (n =42)** | ***χ*^2^** | ***P*** |
| --- | --- | --- | --- | --- | --- |
| ***Medication*** |  |  |  |  |  |
| Antidepressants |  |  |  |  |  |
| SNRI | 6 | 21 | N/A | 11.364 | **<0.001** |
| SSRI | 16 | 11 | N/A | 1.769 | 0.183 |
| Mirtazapine | 6 | 13 | N/A | 2.929 | 0.087 |
| Mood stabilizer | 15 | 1 | N/A | 16.090 | **<0.001** |
| Antipsychotics | 20 | 5 | N/A | 14.030 | **<0.001** |
| Benzodiazepines | 7 | 16 | N/A | 4.306 | **0.038** |
| Monotherapy | 5 | 7 | N/A | 0.285 | 0.594 |
| Unmedication | 4 | 4 | N/A | 0.005 | 0.942 |
| Unclear | 2 | 1 | N/A | 0.374 | 0.514 |

**Note:** two-tailed Chi-square test.

**Abbreviations:** BD-I, bipolar type I depression; UD, unipolar depression; HCs, healthy controls; SNRI, serotonin-norepinephrine reuptake inhibitor; SSRI, selective serotonin reuptake inhibitor.

**Supplemental material S4. Correlation analyses between** **the dReHo variability and** **illness course, depressive/manic/anxiety symptom severity.**

We conducted the correlation analysis between the dReHo variability of brain regions with significant omnibus differences with HAMD, HAMD, YMRS Scores, as well as duration of illness of BD-I and UD, but did not find any significant associations between these parameters. The details were presented in the following **Table S4** and **Figure S1**.

**Table S4.** **Details of Correlation analyses.**

| **Correlative factors** | **The dReHo variability of brain regions with significant differences** | |
| --- | --- | --- |
|  | Right postcentral gyrus | Right parahippocampal gyrus |
| HAMD Scores | **A:** *p* = 0.126; *r* = 0.180 | **B:** *p* = 0.054; *r* = 0.227 |
| HAMA Scores | **C:** *p* = 0.86; *r* = -0.017 | **D:** *p* = 0.876; *r* = 0.019 |
| YMRS Scores | **E:** *p* = 0.377; *r* = 0.105 | **F:** *p* = 0.639; *r =* 0.056 |
| Duration of illness | **G:** *p* = 0.945; *r* = 0.008 | **H:** *p* = 0.784; *r* = 0.033 |


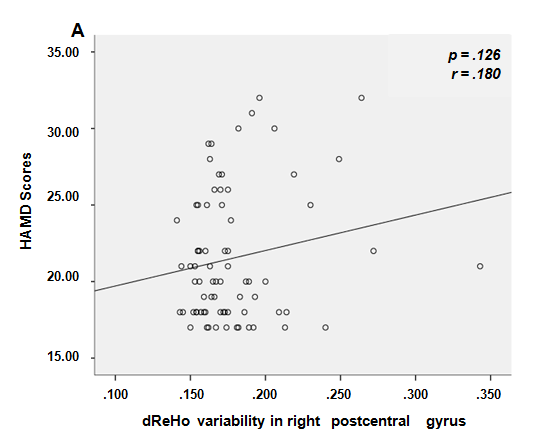

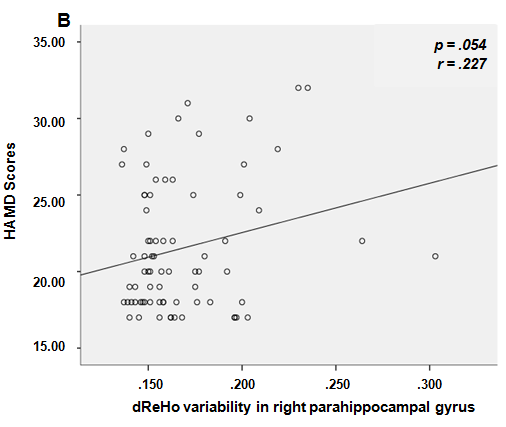


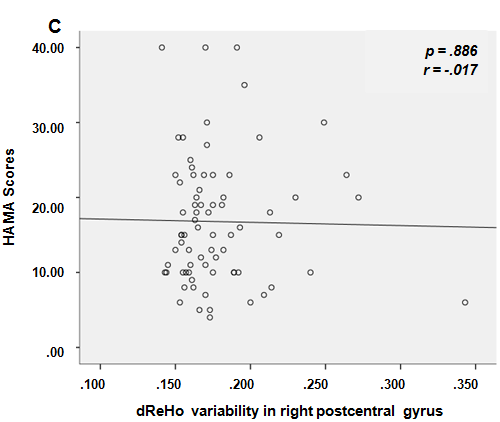

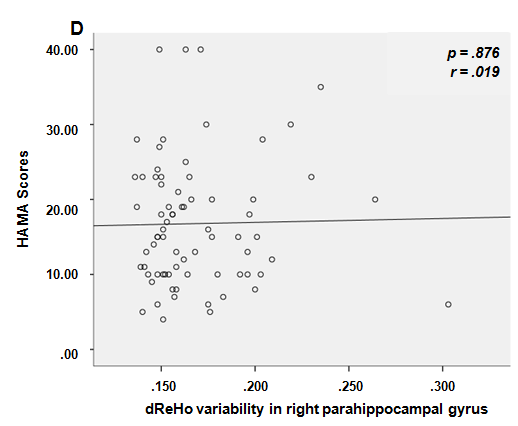


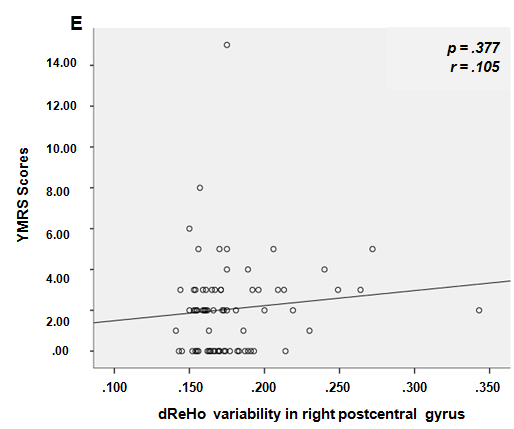

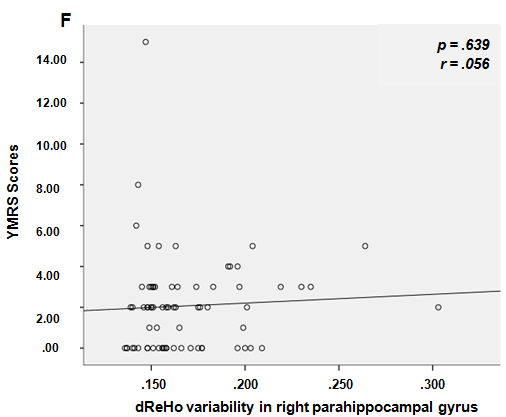


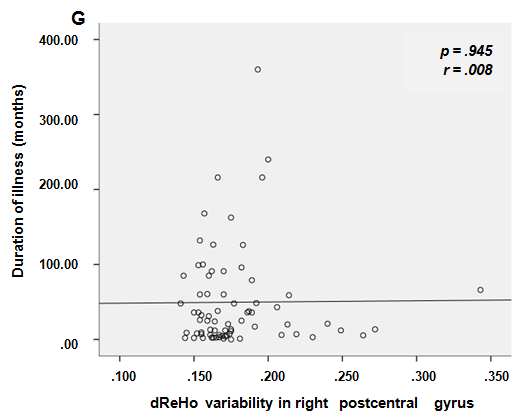

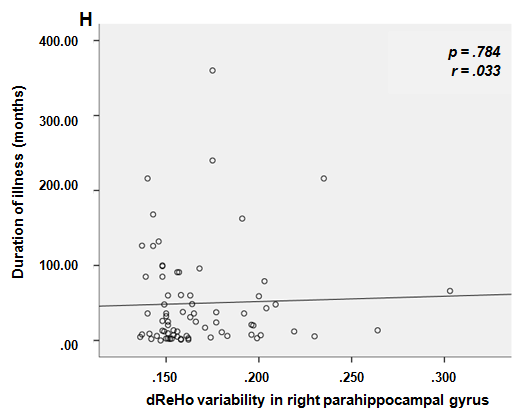


**Figure S1.** **Correlation analyses.**

**Abbreviations**: dReho, the dynamic regional homogeneity; HAMD, Hamilton Depression Rating Scale; HAMA, Hamilton Anxiety Rating Scale; YMRS, Young Mania Rating Scale.
